# A Transposon-Associated CRISPR/Cas9 System Specifically Eliminates both Chromosomal and Plasmid-Borne *mcr-1* in Escherichia coli

**DOI:** 10.1128/AAC.01054-21

**Published:** 2021-09-17

**Authors:** Yu-Zhang He, Jin-Ru Yan, Bing He, Hao Ren, Xu Kuang, Teng-Fei Long, Cai-Ping Chen, Xiao-Ping Liao, Ya-Hong Liu, Jian Sun

**Affiliations:** a National Risk Assessment Laboratory for Antimicrobial Resistance of Animal Original Bacteria, College of Veterinary Medicine, South China Agricultural University, Guangzhou, China; b Guangdong Laboratory for Lingnan Modern Agriculture, South China Agricultural University, Guangzhou, China; c Guangdong Key Laboratory for Veterinary Drug Development and Safety Evaluation, College of Veterinary Medicine, South China Agricultural University, Guangzhou, China; d College of Veterinary Medicine, Southwest University, Chongqing, China

**Keywords:** IS*Apl1*, *mcr-*1, CRISPR/Cas9, transposon, antibiotic resistance

## Abstract

The global spread of antimicrobial-resistant bacteria has been one of the most severe threats to public health. The emergence of the *mcr-1* gene has posed a considerable threat to antimicrobial medication since it deactivates one last-resort antibiotic, colistin. There have been reports regarding the mobilization of the *mcr-1* gene facilitated by IS*Apl1-*formed transposon Tn*6330* and mediated rapid dispersion among *Enterobacteriaceae* species. Here, we developed a CRISPR/Cas9 system flanked by IS*Apl1* in a suicide plasmid capable of exerting sequence-specific curing against the *mcr-1*-bearing plasmid and killing the strain with chromosome-borne *mcr-1*. The constructed IS*Apl1*-carried CRISPR/Cas9 system either restored sensitivity to colistin in strains with plasmid-borne *mcr-1* or directly eradicated the bacteria harboring chromosome-borne *mcr-1* by introducing an exogenous CRISPR/Cas9 targeting the *mcr-1* gene. This method is highly efficient in removing the *mcr-1* gene from Escherichia coli, thereby resensitizing these strains to colistin. The further results demonstrated that it conferred the recipient bacteria with immunity against the acquisition of the exogenous *mcr-1* containing the plasmid. The data from the current study highlighted the potential of the transposon-associated CRISPR/Cas9 system to serve as a therapeutic approach to control the dissemination of *mcr-1* resistance among clinical pathogens.

## INTRODUCTION

Mobile genetic elements (MGEs) such as plasmids, transposons, integrons, and insertion sequences (ISs) play essential roles in disseminating antibiotic resistance genes (ARGs) among Gram-negative bacteria. The colistin resistance gene *mcr-1* was first identified from a conjugative IncI2 plasmid in China and has been intensively reported globally from other plasmid types (1). There have been highly diverse plasmid types involved in the bearing of *mcr-1*, including the IncX4, IncI2, IncP, IncFII, and IncHI2 types (1–4). The IncX4 plasmid has been deemed one of the most prevalent types in Escherichia coli at a prevalence level ranging from 7.6% to 34% (5, 6). Except for the plasmid-borne *mcr-1*, chromosomal copies of *mcr-1* have also been identified in E. coli strains isolated from retail meats and humans (7–9). Notably, a recent study reported an isolate found with three tandem copies of the *mcr-1* element carried by IS*Apl1* (10). The IS*Apl1* belongs to the IS*30* family and was first characterized in Actinobacillus pleuropneumoniae (11). There have been studies demonstrating that IS*Apl1* facilitated the mobilization of *mcr-1* by forming a composite transposon Tn*6330* and preferentially targeting AT-rich sequences (12–14). These results implied the active role of IS*Apl1* in the transposition of *mcr-1* between plasmids and the bacterial chromosome and vice versa.

The clustered regularly interspaced short palindromic repeat (CRISPR)/Cas system was initially discovered as a bacterial immunity in archaea and bacteria against MGEs (15), yet it has now become the predominant technique for gene editing (16). This technique allows an easy-handling approach to knock out, insert, and mutate genes in more than 40 species with high-efficiency and low-cost procedures (17, 18). As to the broad applicability, the CRISPR/Cas9 system showed its potency to eradicate antibiotic resistance genes. Previous studies reported the employment of engineered plasmids to deliver the CRISPR/Cas9 system targeting *mcr-1* in the recipient bacteria (19–21). However, notable demerits for the plasmid-based methods, commonly used in previous reports, are genetic instability and weak control toward the copy numbers of the established systems (22, 23). Alternatively, there have been studies to deliver the CRISPR/Cas9 system with a bacteriophage (24, 25). This strategy could specifically target bacterial genotypes from mixed bacterial populations in both *in vitro* and *in vivo* conditions (25–27), while the limited host ranges and emerging phage resistance might restrain the application of this phage-based strategy. Therefore, developing a novel delivery element for the CRISPR/Cas9 system has been of great scientific significance.

As the principal function of CRISPR/Cas systems in archaea and bacteria is a defense against invasive DNA integration, including viruses, plasmids, and transposons (15). The Tn*7*-like transposons are widely found in the genomes of bacterial and archaeal species and have been proven to recruit CRISPR/Cas systems independently (28). An intriguing example is that the CRISPR/Cas systems work well with Tn*7*-like transposons in targeted transposition, facilitating the spread of genetic elements via phages and plasmids (28, 29). This nature highlighted the potential of a transposon as a tool to deliver the designed CRISPR/Cas systems (29, 30). Thus, we advanced the notion that the transposon-associated CRISPR/Cas system could contribute to the removal of antibiotic resistance. Until now, there has been no report on the development of transposon-associated CRISPR/Cas systems against antibiotic resistance.

**FIG 1:**
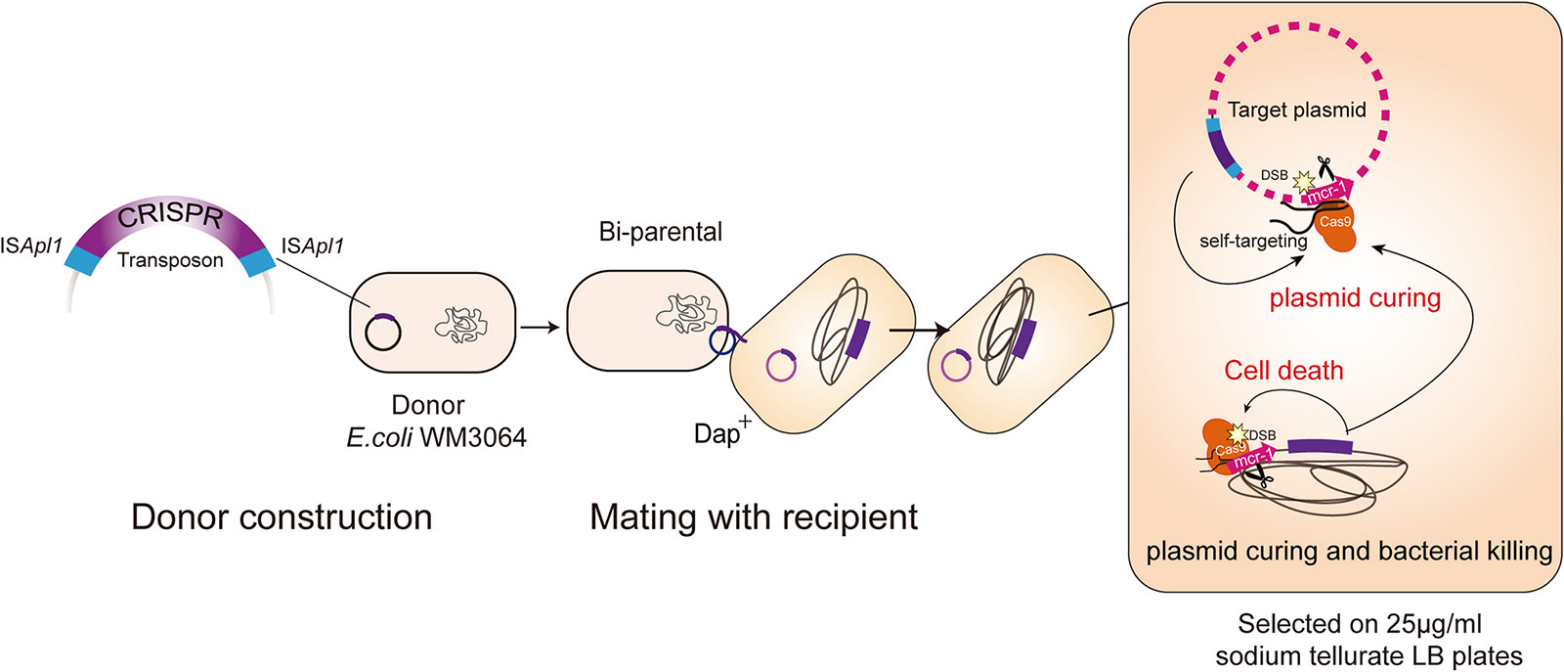
The pIS*Apl1*-CRISPR/Cas9 system for plasmid curing and bacterial killing. A suicide plasmid containing the IS*Apl1*-formed transposon carrying CRISPR/Cas9 was transferred to recipient bacteria by biparental mating. Donor cell E. coli WM3064 contains a chromosomal copy of the RP4 transfer machinery used to mobilize the suicide plasmids. Once inside the recipient cell, The Tn::IS*Apl1*-CRISPR/Cas9 was integrated into the plasmid or chromosome in the recipient strain. Selection on antibiotic plates lacking DAP eliminates the E. coli WM3064 donors and retains recipients with an integrated IS*Apl1*-CRISPR/Cas9 library. The coexpression of sgRNA and Cas9 is capable of plasmid curing and bacterial killing.

ISs and their associated transposons have been greatly reported to mediate the transposition of the resistance genes (31). However, their potential as a carrier to facilitate the CRISPR/Cas system for resistance gene editing and curing in bacteria has been rarely noticed and estimated. In the current study, we employed IS*Apl1* to construct a transposon-associated CRISPR/Cas system as a proof of concept in curing *mcr-1* (Fig. 1). On the one hand, the system successfully eliminates the *mcr-1*-bearing plasmid, even resulting in strains immune from further acquisition of exogenous plasmids carrying *mcr-1*. On the other hand, it could specifically kill the bacteria in which the *mcr-1* is located on the chromosome. This study provides a potent prototype of the transposon-associated CRISPR/Cas system in the fight against colistin resistance. And it might be further employed for the approaches against the antibiotic resistance of other types.

## RESULTS

### Construction of pIS*Apl1*-CRISPR/Cas9 plasmid.

**FIG 2:**
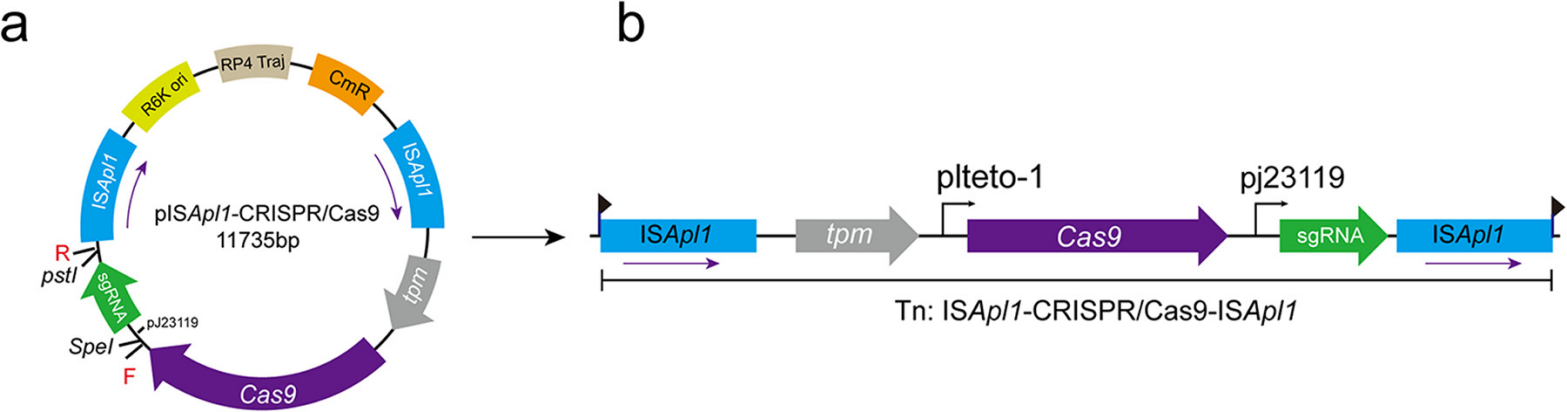
Plasmid map of pIS*Apl1*-CRISPR/Cas9 and the transposon Tn::IS*Apl1*/CRISPR/Cas9. (a) Plasmid pIS*Apl1*-CRISPR/Cas9 containing the *cas9* gene with the promoter pLteto-1 (39) and the sgRNA with the synthetic J23119 promoter; F and R denote the locations of primers used for the sgRNA construct. (b) The Tn::IS*Apl1*-CRISPR/Cas9, the *cas9*, and the *tpm* gene, along with sgRNA was flanked by two IS*Apl1* in the same orientation.

The newly constructed pIS*Apl1*-CRISPR/Cas9 vector contained an R6K replication origin, which relies on the π protein. This suicide plasmid will survive only in bacterial hosts that express the π protein, such as E. coli WM 3064. The Cas9 expression was driven by the pLteto-1 promoter (32), and the single guide RNA (sgRNA) was driven by the constitutive promoter pJ23119. The introduction of the *tpm* gene allowed the selection of clinical multidrug-resistant (MDR) isolates. The recombinant plasmid pIS*Apl1*-CRISPR/Cas9 and the transposon IS*Apl1*-CRISPR/Cas9 are illustrated in Fig. 2.

### Elimination of the *mcr-1* bearing plasmid using pIS*Apl1*-CRISPR/Cas9.

To verify the potency of the IS*Apl1*-CRISPR/Cas system in eliminating the plasmid-borne *mcr-1*, the strains artificially or naturally bearing the *mcr-1* plasmid were used as the model strains. The results showed that the IS*Apl1*-CRISPR/Cas9 was efficiently transferred into the recipient to cut the *mcr-1* harboring plasmids (Table 1), presumably by an unrepairable double-strand break (DSB) caused by highly efficient IS*Apl1*-CRISPR/Cas9 cleavage. The successful elimination of the *mcr-1* was genotypically and phenotypically approved (see Fig. S4 in the supplemental material).

**TABLE 1:** The transposition efficiency of pIS*Apl1*-CRISPR/Cas9 into recipients and the *mcr-1* gene and plasmid curing efficiency^*a*^

Strain	Target gene	Target sequence (N20)	Transposition efficiency (transconjugants/recipient)	Curing efficiency (%)^*b*^
E. coli C600			(5.8 ± 0.6) × 10^−4^	
E. coli C600(pUC19-*mcr-1*)	*mcr-1*	GCGGCATTCGTTATAAGGAT	(7.4 ± 0.5) × 10^−4^	100 ± 0
E. coli CSZ4	*mcr-1*	GCGGCATTCGTTATAAGGAT	(8.9 ± 0.3) × 10^−4^	99.3 ± 1.5
E. coli CSZ4	IncX4 replication gene	AGACTCAAATTCATTGAATC	(6.7 ± 0.8) × 10^−4^	98.5 ± 1.2

a The donor strain used was E. coli WM3064. Selective plating was done on ST25 plates selected for the transconjugants. Transposition efficiency was calculated as the number of transconjugants per recipient.

b The values shown are the mean ± SD from three independent experiments.

### Bactericidal effect of pIS*Apl1*-CRISPR/Cas9 on chromosome-borne *mcr-1* strains.

**FIG 3:**
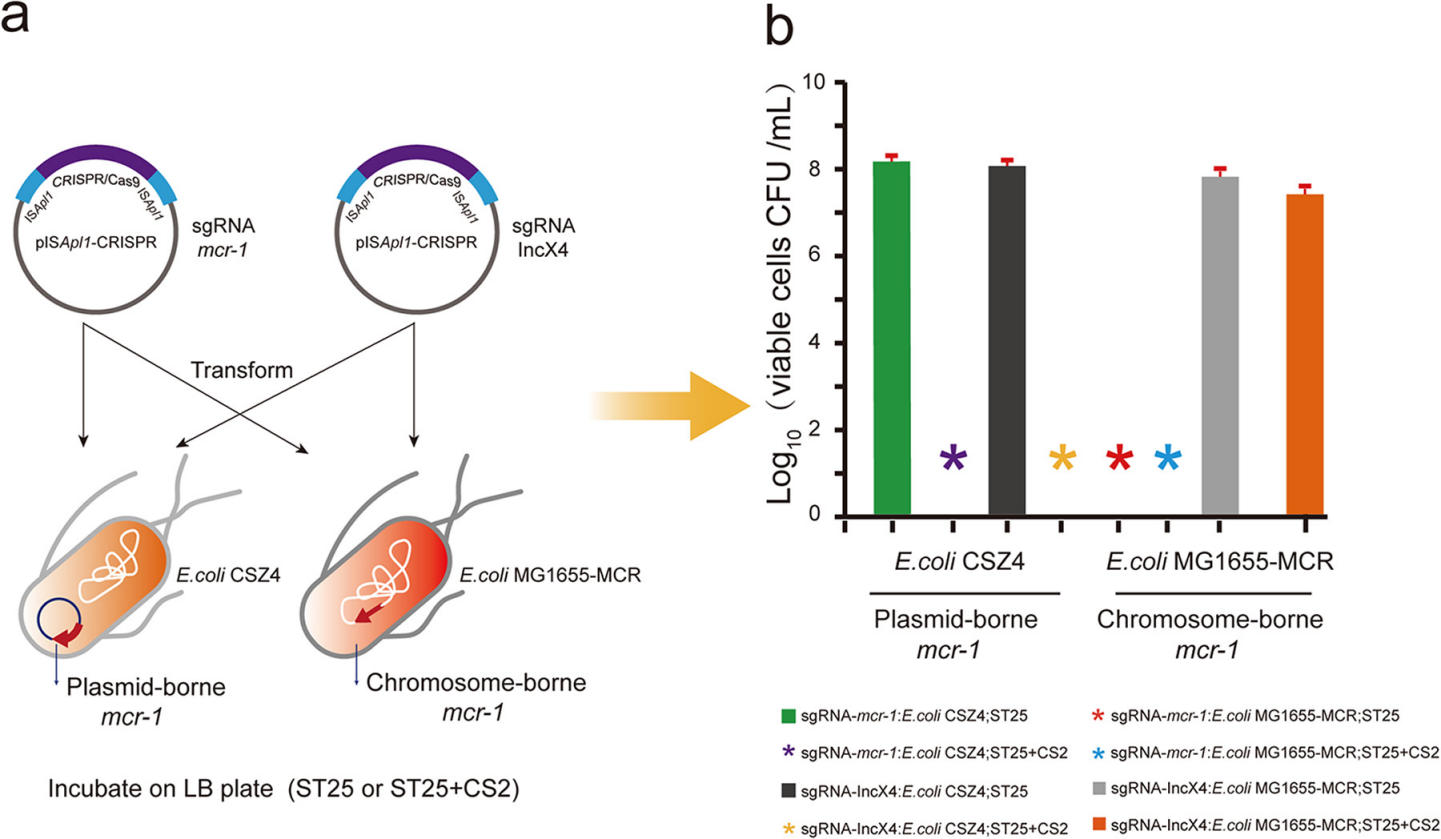
(a) Schematic of mobilizable IS*Apl1*-CRISPR/Cas9-mediated plasmid curing and cell killing. (b) E. coli WM3064 donor cells possessing pIS*Apl1*-CRISPR/Cas9-*mcr-1* or pIS*Apl1*-CRISPR/Cas9-IncX4 were mated at a donor/recipient ratio of 1:1 with strain E. coli CSZ4 harboring a plasmid-borne *mcr-1* or strain E. coli MG1655-MCR in which *mcr-1* is located on the chromosome. Cultures were plated on selective LB agar sodium tellurite (25 μg/ml) in the presence and absence of colistin (2 μg/ml). The mobilization of the pIS*Apl1*-CRISPR/Cas9 construct into cells containing the *mcr-1* gene resulted in plasmid curing and cell death. Mean ± standard error (SE). Asterisks denote the absence of detectable transconjugant colonies.

In this section, we further applied the IS*Apl1*-CRISPR/Cas9 system to the strain that harbored the chromosome-borne *mcr-1*. Impressively, a very remarkable reduction in the viability of *mcr-1*-positive cells was observed as no detectable colonies on the selective agar. However, the IS*Apl1*-CRISPR/Cas9(sgRNA::IncX4) exerted no impact on cell viability (Fig. 3). The system was supposed to cut the chromosome at the site of *mcr-1* and then lead to cell death of *mcr-1*-positive bacteria. The results suggested the specificity of the system with high efficiency and accuracy.

### The integrated IS*Apl1*-CRISPR/Cas9 system blocks plasmid acquisition.

**FIG 4:**
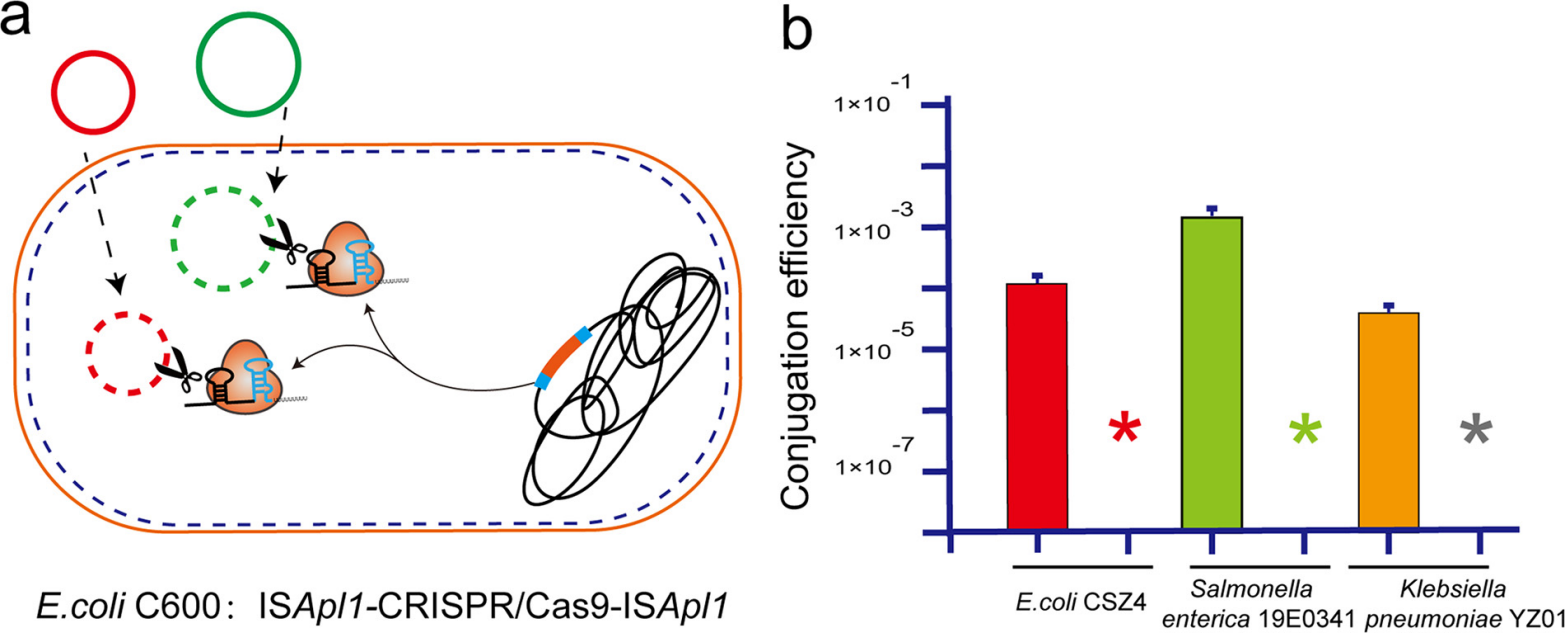
(a) Schematic diagram of the immune system against foreign plasmids of E. coli C600 with IS*Apl1*-CRISPR/Cas9 integrated into the chromosome. (b) The strain E. coli C600 with the chromosome bearing IS*Apl1*-CRISPR/Cas9-IS*Apl1* and the parental strain E. coli C600 were used as recipients conjugated with donor strains E. coli CSZ4, S. enterica 19E0341, and K. pneumoniae YZ01, respectively. The bar chart represented the results when E. coli C600 was used as a recipient (positive control). The asterisks denote the chromosome-borne IS*Apl1*-CRISPR/Cas9-IS*Apl1* strain used as recipient. Each experiment was performed in triplicate. Data points represent the mean values of three biological replicates with error bars showing standard deviation (SD).

The CRISPR/Cas system conferred adaptive immunity against the incorporation of exogenous genetic elements in many bacteria and most archaea. We, therefore, examined whether an integrated CRISPR/Cas9 system was capable of precluding the acquisition *mcr-1*. The plasmids bearing *mcr-1* from E. coli CSZ4, Salmonella enterica 19E0341, and Klebsiella pneumoniae strain YZ01 were conjugated into the pUC19-*mcr-1*-cured strain, E. coli C600, which contained the integrated transposon IS*Apl1*-CRISPR/Cas9(sgRNA::*mcr-1*). As shown in Fig. 4, genetic incorporation of IS*Apl1*-CRISPR/Cas9 curtailed the ARG transfer to the strains yet did not acquire any of the donor strain plasmids, whereas the control E. coli C600 demonstrated particularly high frequencies of *mcr-1* acquisition.

### Escape mutant analysis.

**FIG 5:**
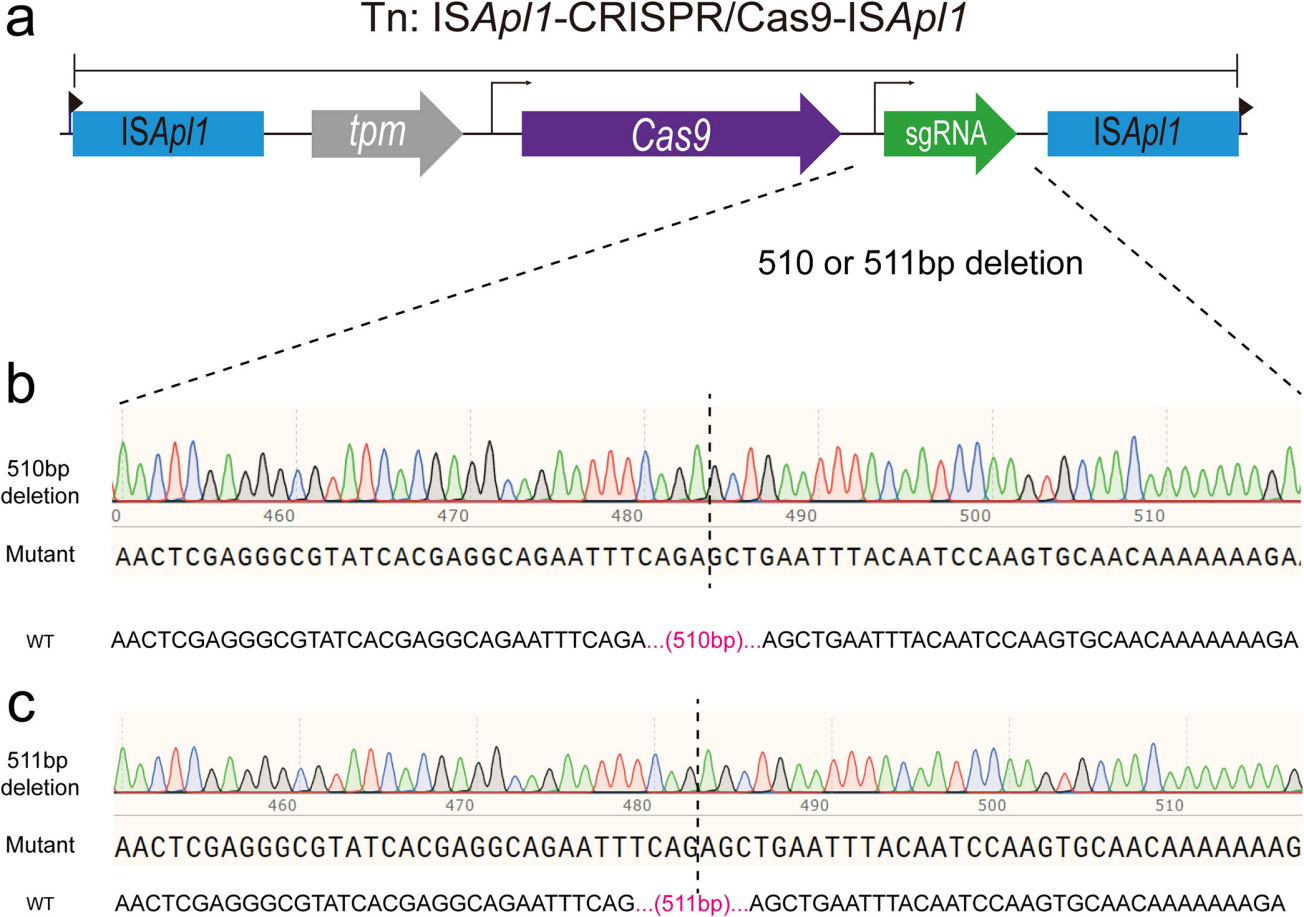
Characterization of escape mutants that tolerated transformation of the pIS*Apl1*-CRISPR/Cas9 construct. (a) The CRISPR/Cas9 region of the escape mutants was amplified by a high-fidelity enzyme and followed by sanger sequencing. (b, c) The results show that 510 or 511 deletions at the same sgRNA area led to pISApl1-CRISPR/Cas9 inactivation in the successful transformants.

A handful of cells of E. coli CSZ4 revealed colistin resistance after receiving the IS*Apl1*-CRISPR/Cas9(sgRNA::*mcr-1*) system. We, therefore, analyzed their *cas9* along with the sgRNA region by PCR. The Sanger sequencing results showed 5 of them with a 510-bp deletion, while 3 demonstrated another 511-bp deletion that includes the sgRNA (Fig. 5).

## DISCUSSION

The occurrence and dissemination of colistin resistance gene *mcr-1* in bacteria severly challenged the current treatment of clinical infections. Therefore, it is essential to develop new methods and strategies to tackle the distribution of *mcr-1*, and the restoration of bacterial sensitivity to colistin would be of great scientific significance. Transposons (Tn) and insertion sequences (IS) are often associated with the transmission of ARGs. They are discrete DNA segments that are able to move their flanked sequences and themselves *per se* randomly to new locations in identical or different DNA (31). In our previous study, we showed that an IS*Apl1*-formed transposon Tn*6330* mediated the *mcr-1* gene transposition at an exceptionally high level (12). This also corresponded to the investigation of Laurent Poirel and colleagues (14), indicating that the ISs and associated Tns might be powerful tools to expedite the CRISPR/Cas editing in bacterial species. Based on our research foundation of IS*Apl1*, we developed an IS*Apl1*-based transposon carrying the CRISPR/Cas9 system to combat the dispersion of *mcr-1*. Once introduced into the targeted strains, the IS*Apl1*-CRISPR/Cas9 cassette randomly integrated into either the chromosome or plasmid or even both in some cases. Current antibiotics tend to be broad-spectrum, leading to indiscriminate killing of commensal bacteria and stimulating drug resistance evolution (24). In our study, the sgRNA was designed to guide the Cas9 protein specifically to the site of *mcr-1* and induce DSBs. The RNA-guided nuclease (RGN) targeting this resistance gene is delivered efficiently using IS*Apl1*-CRISPR/Cas9 carrying suicide plasmids by conjugation. It introduced the DBSs to the DNA segment involving the *mcr-1* gene specifically, making it barely possible to result in indiscriminate killing. Considering that some strains carry *mcr-1* on their chromosomes, we sought to determine the impact of the constructed system on strains with chromosome-harbored *mcr-1*. The results indicated that the system mediated direct killing in the strains of the type as mentioned above (Fig. 3b). This implied that RNA-guided nucleases (RGNs) target specific DNA sequences regardless of whether they are on plasmid or chromosome.

One nature of the translocation of the transposon is its random integration into the bacterial genome. This feature leads to nonmarkerless deletion, which means it is impossible to remove the CRISPR/Cas9 elements from the strains that receive editing. However, given the fact that the CRISPR/Cas system is an adaptive immune system of bacteria and archaea against foreign DNA, the genomically incorporated IS*Apl1*-CRISPR/Cas9 system may confer bacterial immunity from further acquisition of eliminated DNAs. Here, we present an interesting example that the employment of transposon-associated CRISPR/Cas9 offered the recipients stable resistance to foreign integration of *mcr-1* from other bacteria. This “vaccine-like” effect may provide a robust scientific approach for controlling the transmission of the *mcr-1* gene. In CRISPR/Cas editing, the off-target property of the system has been a bottleneck for its successful application (33). In our study, we demonstrated the sequence-specific removal of *mcr-1*-harboring strains using a transposon-associated CRISPR/Cas9 system. We observed highly efficient removal of the *mcr-1* gene (>98% as shown above) and promising specificity by the system. However, a small number of the transformed cells of E. coli CSZ4 remained colistin resistant after CRISPR/Cas9 editing. Sequencing of these survivors revealed consistent loss of sgRNA targeting in the transformed transposon IS*Apl1*-CRISPR/Cas9. This insight is consistent with the findings of a previous study showing loss or inactivation of CRISPR elements under evolutionary pressure (34).

Compared to the several previous studies that require specific reagents (e.g., arabinose, anhydrous tetracycline, rhamnose) to induce the CRISPR/Cas9 system to cure the antibiotic resistance genes (19, 20, 35, 36), the system established in the current study enjoys the benefit of autonomous transposition and the self-driven function of CRISPR/Cas editing, which is particularly meaningful for its *in vivo* applications. Since the concentrations of the inducing reagents are hard to control in the *in vivo* conditions, the self-driven expression of Cas9 will be ideal in practical applications. There is an argument that the continuously expressed Cas9 protein will limit the transformation efficiency of the CRISPR/Cas-containing plasmids. Fortunately, the IS*Apl1*-CRISPR/Cas9 system in this study maintained a high level of transposition efficiency. This is probably because IS-formed transposon partially compensates for the efficiency reduction. To conclude, the CRISPR/Cas9 system mobilized by transposon to exclude antibiotic resistance genes was first established in the present study. This novel strategy demonstrated substantially high efficiency in removing *mcr-1* genes from clinical or lab *mcr-1*-positive strains and kill the strains with chromosomal *mcr-1* genes. Moreover, the integration of the CRISPR/Cas9 system conferred immunity to the cured bacteria from the further acquisition of *mcr-1* genes. With the efficiency and safety observed, the reported system showed great potential in MDR bacteria treatment and expanded the arsenal of tools against a possible superbug crisis.

## MATERIALS AND METHODS

### Bacterial strains.

The strains used in this study are listed in Table 2, and primers can be found in Table S1 in the supplemental material. E. coli strain WM3064 was used as the donor strain in the study. All strains were cultured at 37°C in Luria Bertani (LB) medium, and diaminopimelic acid (DAP) was supplemented at 0.3 mM for the conjugation assays. When necessary for the selection of transformants, the antibiotics were added at the following concentrations: colistin at 2 μg/ml (abbreviated to CS2), chloramphenicol at 25 μg/ml (abbreviated to C25), and sodium tellurite at 25 μg/ml (abbreviated to ST25).

**TABLE 2:** Plasmids and strains used in this study

Strain or plasmid	Description	Reference or study
Strain		
E. coli C600	Conjugation recipient, F^−^ *tonA21 thi-1 thr-1 leuB6 lacY1 glnV44 rfbC1 fhuA1* λ^−^	40
E. coli C600(pUC19-*mcr-1*)	E. coli C600 harboring pUC19-*mcr-1*	This study
E. coli CSZ4	Clinical isolate harboring an IncX4 plasmid pCSZ4 bearing *mcr-1*	1
E. coli MG1655-MCR	E. coli MG1655(Δ*recA*::Kan), chromosome bearing *mcr-1*	12
Salmonella enterica 19E0341	Clinical isolate harboring an IncI2 plasmid bearing *mcr-1*	41
Klebsiella pneumoniae YZ01	Clinical isolate harboring an IncI2 plasmid bearing *mcr-1*	This study
Plasmid		
pIS*Apl1*-CRISPR/Cas9	Suicide plasmid harboring Tn::IS*Apl1*-CRISPR/Cas9, R6K *ori*, *mobRP4*	This study
pUC19-*mcr-1*	pUC19 derivative harboring *mcr-1*	This study

### Plasmid construction.

We constructed a suicide plasmid consisting of the CRISPR/Cas9 cassette flanked by IS*Apl1*, which possessed the RP4*_oriT_* fragment from pCVD442 (37) and the R6K replication origin from pSV03 (12). Traditional antibiotic selection markers were replaced by the Acinetobacter baylyi-derived tellurite resistance marker gene *tpm*, encoding a thiopurine *S*-methyltransferase, to allow selection for chromosomal plasmid integration with sodium tellurite (38). Plasmid construction is described in greater detail in Appendix S1 in the supplemental material.

### sgRNA design and cloning.

A 20-nt base-pairing region (N20) of an sgRNA was designed using the Custom Dicer-Substrate siRNA (DsiRNA) tool (https://eu.idtdna.com/site/order/designtool/index/crispercustom). The sgRNA fragments with N20 were amplified with primers AATACTAGT-N20- GTTTTAGAGCTAGAAATAGC and GGACTGCAGGCAACGTTCAA (restriction sites are underlined) using pIS*Apl1*-CRISPR/Cas9 plasmid as the template. The PCR products were successively ligated to predigested pIS*Apl1*-CRISPR/Cas9 plasmid at the SpeI and PstI sites, generating pIS*Apl1*-CRISPR/Cas9 plasmids containing sgRNAs specific for *mcr-1* and IncX4. The cloning procedure of the targeted sgRNA into the pIS*Apl1*-CRISPR/Cas9 was shown in Fig. S3 in the supplemental material.

### Transposition assays.

The transposition was evaluated by the biparental mating assay with the pIS*Apl1*-CRISPR/Cas9-bearing E. coli WM3064 as the donor. In brief, donor strains were grown overnight at 37°C in LB supplemented with 300 μM DAP, and recipients were cultivated until the early stationary phase. Donors and recipients (100 μl of each) were combined in 700 μl LB and then centrifuged for 2 min at 7,000 × *g*. The cell pellets were washed with phosphate-buffered saline (PBS) three times and then resuspended in 50 μl LB. The mixture was, after that, transferred onto an MF HAWG01300 cellulose membrane (Millipore, Burlington, MA, USA) and placed on the prewarmed LB agar and incubated at 37°C for 12 h. The transconjugants were liberated from the membrane by vortexing in PBS and then serially diluted and plated on ST25 LB and antibiotic-free LB agar plates to calculate the transposition efficiency. To calculate the viable cell CFU of the plasmid-harboring *mcr-1* and chromosome-harboring *mcr-1* strains, the transconjugants were then serially diluted and plated on ST25 LB agar plates or CS2+ST25 LB agar plates.

### *In vitro* plasmid curing efficiency evaluation.

The strains E. coli CSZ4 and E. coli C600(pUC19-*mcr-1*), which naturally or artificially harbored the *mcr-1* gene and the E. coli C600 strain, were used in the conjugation assay to assess the plasmid curing and transposition efficiency of pIS*Apl1*-CRISPR/Cas9-*mcr-1*. To confirm the loss of the pCSZ4 and pUC19-*mcr-1* in the successful transformants, fifty colonies grown on ST25 plates were randomly selected and subjected to PCR detection using primers IncX4-TF+IncX4-TR and mcr-TF+mcr-TR targeting the replication gene and *mcr-1* gene of pCSZ4, respectively, and the primers UC19-TF+UC19-TR and mcr-TF+mcr-TR targeting the replication gene and *mcr-1* gene of pUC19-*mcr-1*, respectively. The plasmid curing efficiency was calculated based on the PCR detection results. The experiments were technically repeated twice with three biological replicates.

### *In vitro* sequence-specific bacterial killing using pIS*Apl1*-CRISPR/Cas9.

To test the bacterial killing capacity of IS*Apl1*-CRISPR/Cas9, the pIS*Apl1*-CRISPR/Cas9 targeting *mcr-1* was introduced into E. coli MG1655-MCR by conjugation, and the viable cells after treatment were calculated from the ST25 or ST25+CS2 agar plates. The experiments were technically repeated twice with three biological replicates.

### Sequencing of escape mutants.

For plasmid curing, the transformants spread on LB agar plates with ST25 were verified by genotypes and phenotypes. Through PCR, we noticed that a minimal number of colonies were positive and that these colonies could grow on the agar plate of CS2. Then genomic DNA was extracted from escape mutants using a TIANamp bacteria DNA kit (Tiangen, Beijing, China). The Cas9+sgRNA containing regions of each escape mutant were amplified using primers mut-Cas9F and mut-Cas9R and subjected to DNA sequencing.

### The IS*Apl1*-CRISPR/Cas9 system preventing plasmid acquisition.

In this section, we used the plasmid-cured strain E. coli C600(IS*Apl1*-CRISPR/Cas9) as the recipient in conjugation assay to see whether the integration of IS*Apl1*-CRISPR/Cas was able to impede the acquisition of *mcr-1* from model strains, and the donors were E. coli CSZ4, Salmonella enterica 19E0341, and Klebsiella pneumoniae YZ01, respectively. The conjugation assay was performed as described above. The blockade of *mcr-1* transfer was evaluated by conjugation efficiency. The experiments were twice technically repeated with at least three biological replicates.

### Accession number(s).

The complete nucleotide sequence of pIS*Apl1*-CRISPR/Cas9 was deposited in GenBank under accession number MW811192.
