# Supplementary material for: A Transposon-Associated CRISPR/Cas9 System Specifically Eliminates both Chromosomal and Plasmid-Borne mcr-1 in Escherichia coli
Source: Antimicrob Agents Chemother. 2021 Sep 17;65(10):e01054-21. doi: 10.1128/AAC.01054-21 (PMC8448152; doi:10.1128/AAC.01054-21)
Supplement: Supplemental file 1 — Supplemental material. Download AAC.01054-21-s0001.pdf, PDF file, 13.5 MB [file aac.01054-21-s0001.pdf]

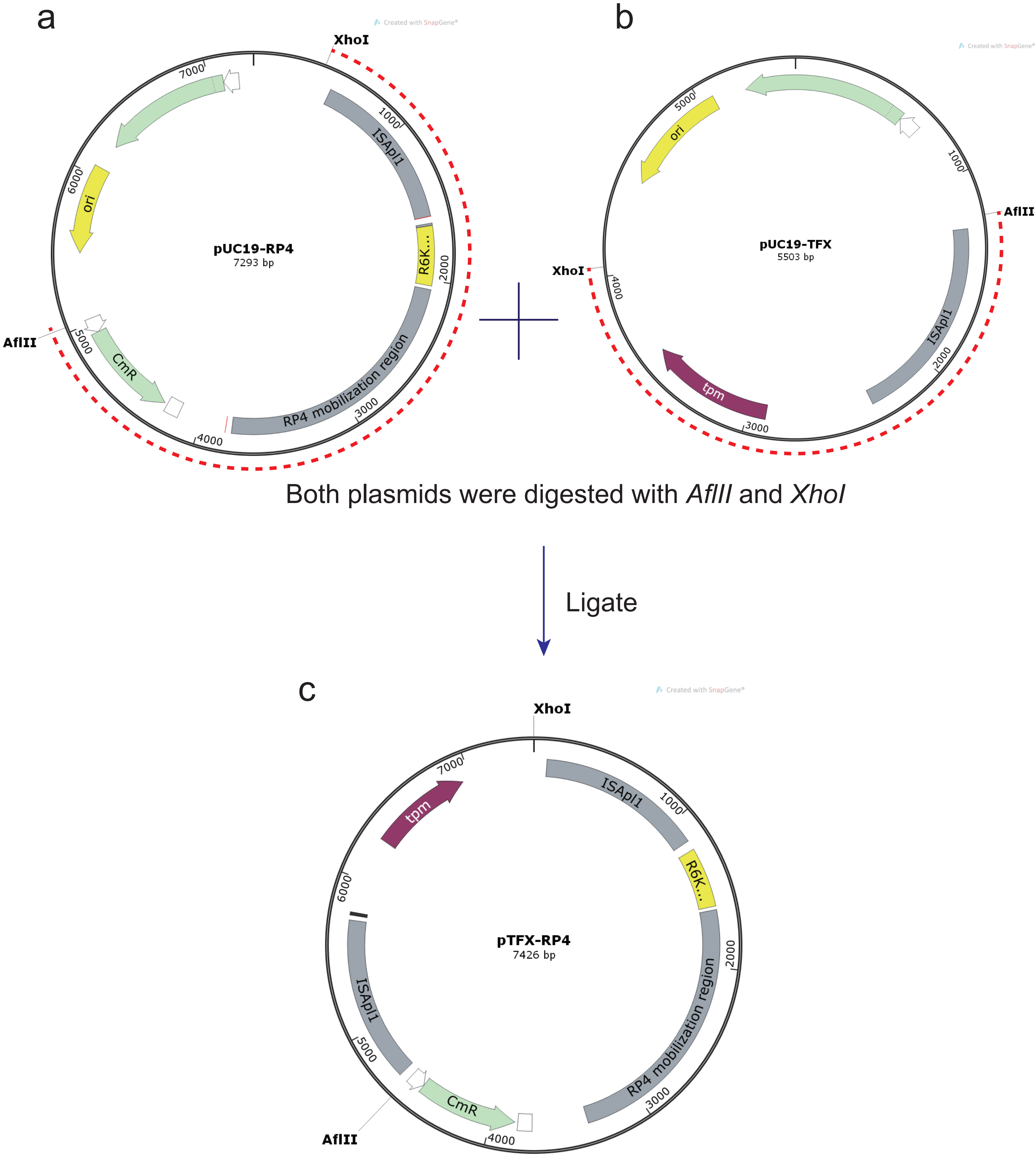
a
XhoI
7000
1000
ISApl1
6000
ori
pUC19-RP4
7293 bp
R6K...
2000
AflII
5000
CmR
RP4 mobilization region
3000
4000
b
5000
ori
1000
AflII
XhoI
pUC19-TFX
5503 bp
4000
ISApl1
2000
tpm
3000
Both plasmids were digested with *AflII* and *XhoI*
Ligate
c
XhoI
7000
ISApl1
1000
tpm
R6K...
6000
pTFX-RP4
7426 bp
2000
ISApl1
RP4 mobilization region
5000
CmR
3000
AflII
4000

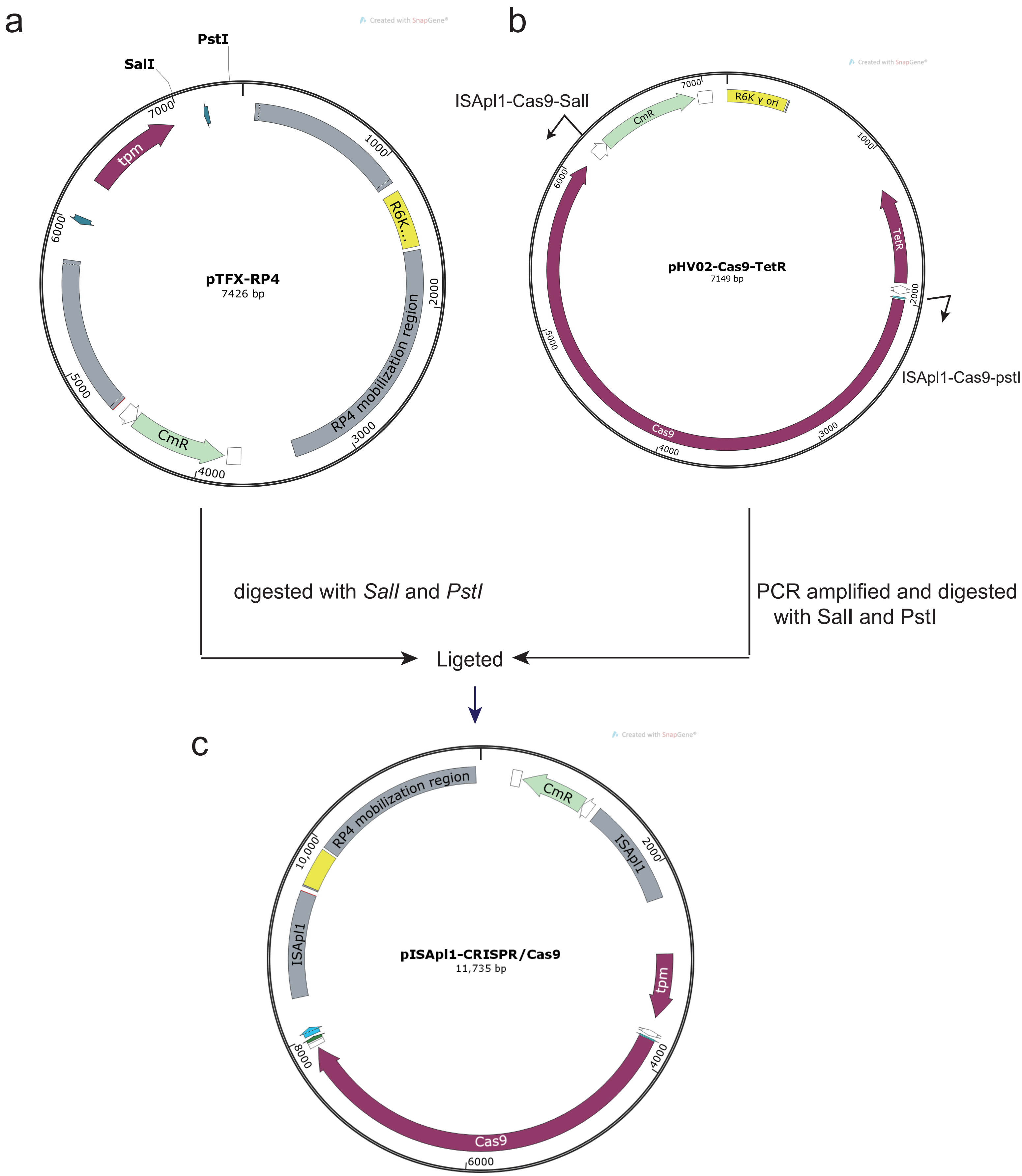
a
SalI
PstI
tpm
R6K...
RP4 mobilization region
CmR
pTFX-RP4
7426 bp
1000
2000
3000
4000
5000
6000
7000
b
ISApl1-Cas9-SalI
ISApl1-Cas9-pstI
CmR
R6K γ ori
TetR
Cas9
pHV02-Cas9-TetR
7149 bp
1000
2000
3000
4000
5000
6000
7000
digested with *SalI* and *PstI*
PCR amplified and digested with SalI and PstI
Ligeted
c
RP4 mobilization region
CmR
ISApl1
ISApl1
tpm
Cas9
pISApl1-CRISPR/Cas9
11,735 bp
2000
4000
6000
8000
10,000

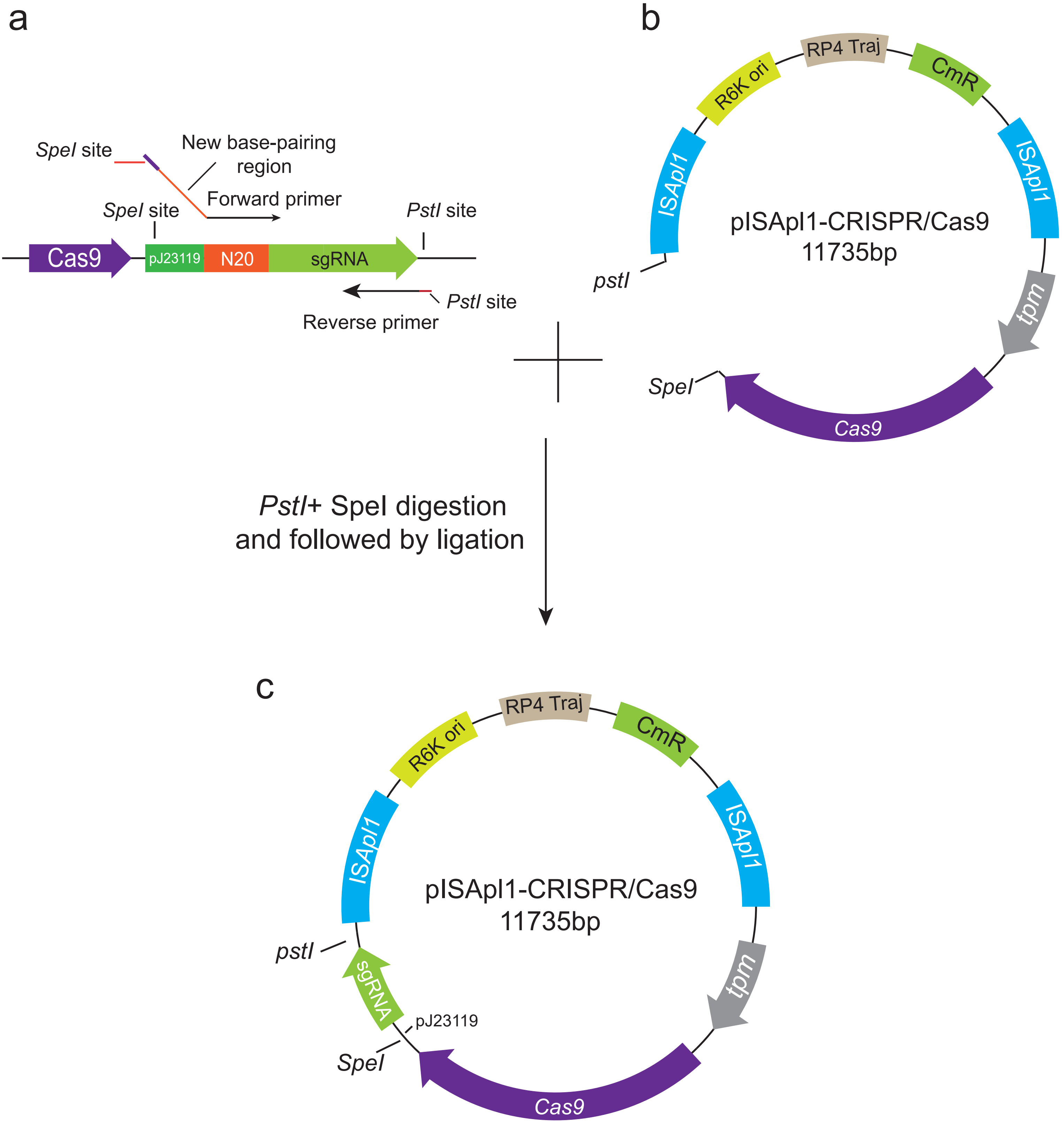

a
SpeI site
New base-pairing region
Forward primer
SpeI site
PstI site
Cas9
pJ23119
N20
sgRNA
PstI site
Reverse primer
b
RP4 Traj
R6K ori
CmR
ISApl1
ISApl1
pISApl1-CRISPR/Cas9
11735bp
pstI
tpm
SpeI
Cas9
PstI+ SpeI digestion
and followed by ligation
c
RP4 Traj
R6K ori
CmR
ISApl1
ISApl1
pISApl1-CRISPR/Cas9
11735bp
pstI
sgRNA
tpm
pJ23119
SpeI
Cas9

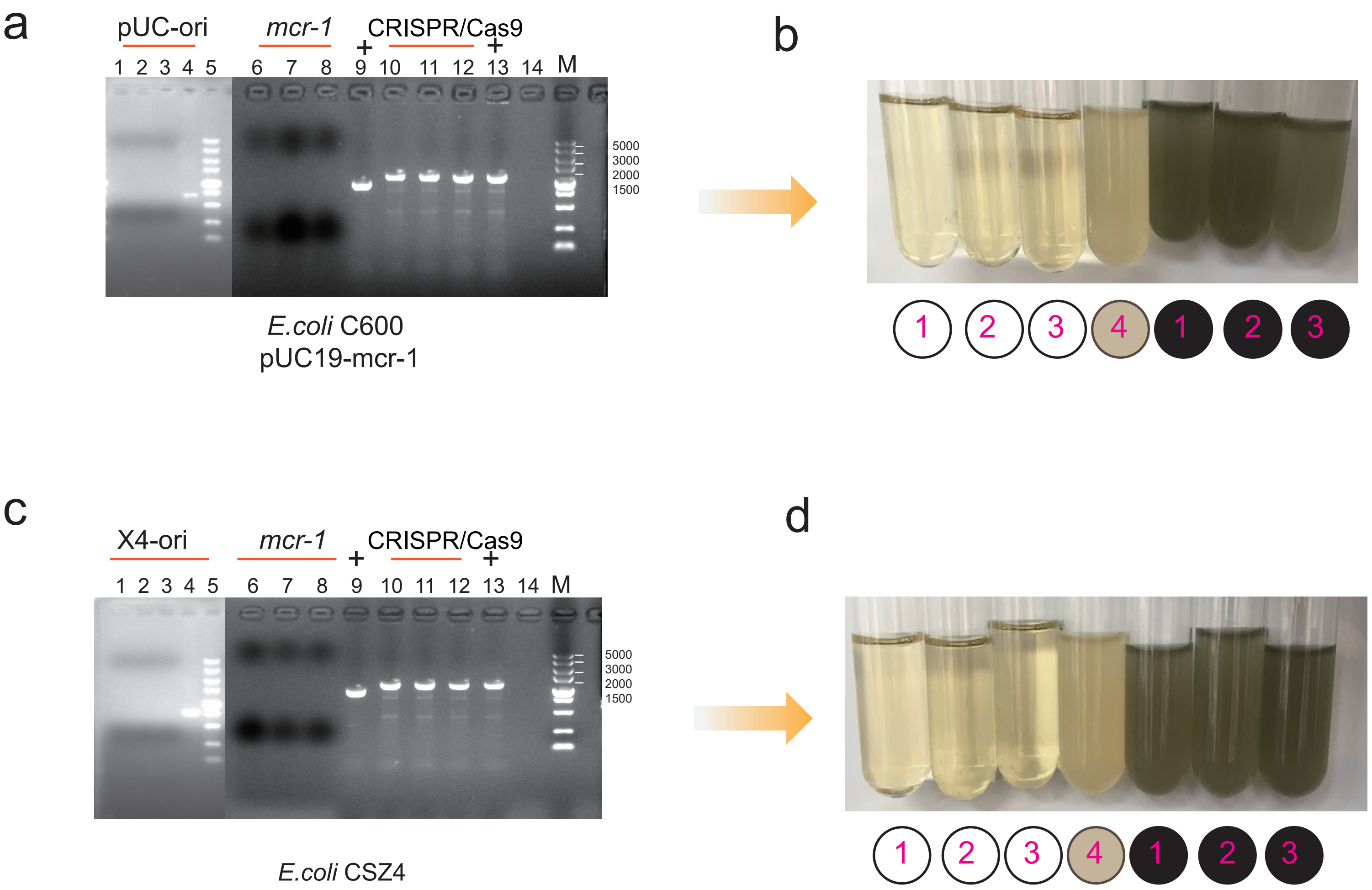
a
pUC-ori
mcr-1
CRISPR/Cas9
+ +
1 2 3 4 5 6 7 8 9 10 11 12 13 14 M
5000
3000
2000
1500
E.coli C600
pUC19-mcr-1
b
1 2 3 4 1 2 3
c
X4-ori
mcr-1
CRISPR/Cas9
+ +
1 2 3 4 5 6 7 8 9 10 11 12 13 14 M
5000
3000
2000
1500
E.coli CSZ4
d
1 2 3 4 1 2 3

Supplementary materials

Table S1; Primers used in this study

Primers	Sequences (5′-3′)	References
RP4-F	GGTGAGAATCCAAGCACTAGCTATCGCTACGTGACTGGGT	This study
RP4-R	CCATGTCAGCCGTTAAGTGT	This study
IS-F1	AGCTCGGTACCCGGGGATCCTACGCCTCGAGTGGTGTCGATGATGCTTGCG	This study
IS-R1	CTTTCGCTAAGGATGATTTCGGCCTAGGCGGCCACACCATAATGCCCAGTTCC	This study
CmR-F	CCAAGCTTGCATGCCTGCAGTACGCCTTAAGTGATCGGCACGTAAGAGGTT	This study
CmR-R	CTAGTGCTTGGATTCTCACC	This study
UC19-F1	CTGCAGGCATGCAAGCTTGG	This study
UC19-R1	GGATCCCCGGGTACCGAGCTCGAATTCACTGGCCGTCGTTTTAC	This study
TPM-HF1	GTGTATATTCAGTATGGGATTGCG	This study
TPM-HR1	CGGTGGAATCGAAATCTCGTGCCATGCATGTCGACTCTAG	This study
IS-F2	CCTAGGCCTGGAGATCTACTACTGTGGCTAAGCCTCAACTTA	This study
IS-R2	CGCAATCCCATACTGAATATACAC	This study
UC19-F2	ACGAGATTTCGATTCCACCG	This study
UC19-R2	AGTAGATCTCCAGGCCTAGG	This study
SpeI-N20-mcr	AATACTAGTGCGGCATTCGTTATAAGGATGTTTTAGAGCTAGAAATAGCAAGTTAAAATAAGGC	This study
SpeI-N20-IncX4	AATACTAGTAGACTCAAATTCATTGAATCGTTTTAGAGCTAGAAATAGCAAGTTAAAATAAGGC	This study
ISApII-Cas9-salI	TAAGTCGACGCAGCTCTAATGCGCTGTTA	
ISApl1-sgRNA-pstI	TAACTGCAGGCAACGTTCAAATCCGCTCC	This study
mut-Cas9F	GCAGCTCTAATGCGCTGTTAATC	This study

mut-Cas9R	TGTTTTTTGCCCTGTGCGAGTA	This study
mcr-TF	GATCACCACGCTGTTATCATCG	This study
mcr-TR	CATATCTTTGCCGTTATTGGCAGC	This study
UC19-TF	TTTCCATAGGCTCCGCCCC	This study
UC19-TR	TTGAGATCCTTTTTTTCTGCGCG	This study
IncX4-TF	CTCTGAGTCTTCCCTGGTATCG	This study
IncX4-TR	CAACATTGCCTATGGCTGCT	This study

Appendix 1

The construction of pISApl1-CRISPR/Cas9

In brief, the plasmid was constructed by first generating plasmid pUC19-RP4 (**FigureS1 a)** that contained the RP4oriT conjugation transfer fragment. The R6K ori was amplified using primers RP4-F and RP4-R from pCVD442(1). A single copy of IS*Apl1* was amplified from the genome of *E. coli* strain FS13Z2S (2) using primers IS-F1 and IS-F2. The chloramphenicol resistance gene CmR was amplified from pSV03(3) using primers CmR-F and CmR-R, and the backbone was amplified from pUC19 using primers UC19-F1 and UC19-R1. These four DNA fragments with homologous sequences at their ends were used to generate pUC19-RP4 by homologous recombination using the NEBuilder HiFi DNA Assembly kit (New England Biolabs, Beverley, MA, USA). Then, a synthetic cassette containing the thiopurine S-methyltransferase (*tpm*) gene driven by the rpsL PCS12 promoter (4) that was cloned into pKD4(5) to generate pKD4-tpm. The *tpm* gene was then amplified using primers TPM-HF1 and TPM-HR1. Another single copy of IS*Apl1* that differed from the one contained in pUC19-RP4 was amplified from the genome of *E. coli* FS13Z2S using primers IS-F2 and IS-R2 and the plasmid backbone was amplified from pUC19 using primers UC19-F2 and UC19-R2 as above. These four DNA fragments were assembled by homologous recombination to generate pUC19-TFX (**FigureS1 b**). The pTFX-RP4 plasmid was constructed by digesting pUC19-RP4 and pUC19-TFX plasmids with *AflII* and XhoI and the digested fragments (The red dotted line **FigureS1 a b**) were purified from agarose gels. The purified fragments were ligated giving rise to the recombinant plasmid pTFX-RP4 (**FigureS1 c**).

The Cas9 gene and sgRNA from pHV02-Cas9 (Laboratory stock, **Figure S2b**) were subsenquently amplified with primers Cas9-SalI and Cas9-PstI as previously described. The amplicons were then digested and ligated to plasmid pTFX-RP4 at the same restriction sites, resulting in the recombinant plasmid pIS*Apl1*-CRISPR/Cas9 (**Figure S2c**).

Figure Lengens

Figure S1;**(a)** The plasmid map of the pUC19-RP4, **(b)** The plasmid map of the pUC19-TFX. **(c)**The plasmid map of the pTFX-RP4.

Figure S2;(a) Construction of pIS*Apl1*-CRISPR/Cas9. (a)The map of the pTFX-RP4, (b)The plasmid map of the pHV02-Cas9-TetR. (c)The plasmid map of pIS*Apl1*-CRISPR/Cas9.

Figure S3; The new N20 was cloned by PCR using the pIS*Apl1*-CRISPR/Cas9 as a template. The *pstI* and *SpeI* were located on the promoter and downstread of the sgRNA, respectively, to facilitate sgRNA cloning. **(b)**The *pstI* and *SpeI* digested pISApl1-CRISPR/Cas9 was used as a backbone to clone the new sgRNA. **(c)**The pIS*Apl1*-CRISPR/Cas9 with new targeted sgRNA.

Figure S4; PCR and Phenotypic confirmation of colistin-sensitive mutants generated from *mcr-1* as a sgRNA target in *E. coli* C600(pUC19-mcr-1) and *E. coli* CSZ4. **(a, c)** Lane1-3 was amplified using the specific primers UC19-TF, UC19-TR, and IncX4-TF, IncX4-TR to test the replication gene of the pUC19-mcr-1 and pCSZ4 in the plasmid cured strain *E. coli* C600(pUC19-mcr-1), *E. coli* CSZ4, respectively. Lane 4 was the positive control. Lane 6-8 was amplified using the specific primers mcr-TF, mcr-TF to test the *mcr-1* gene of the plasmid cured strain *E. coli* C600(pUC19-mcr-1), *E. coli* CSZ4, respectively. Lane 9 was the positive control that the *mcr-1* gene was used as a template. Lane 10-12 was amplified using the specific primers LHCas9TF, ISApl1-F to test the ISApl1-CRISPR/Cas9 system, Lane 13 was the positive control that the pISApl1-CRISPR/Cas9 was used as a template. **(b, d)** phenotypic validation of the plasmid cured strain *E. coli* C600(pUC19-mcr-1) and *E. coli* CSZ4, respectively. ①② ③represents the above mentioned Lane1-3, 6-8,10-12 PCR colonies grow in LB barth supplemented with 2μg/ml colistin and 25μg/m sodium tellurite. ④represents the parent strain *E. coli* C600(pUC19-mcr-1) and *E. coli* CSZ4 grow in LB barth supplemented with 2μg/ml colistin. The numbers 1-3, 6-8, and 10-12 denote three individual repeats of overnight cultures of plasmid cure strains were diluted 100-fold and used as a PCR template

References

1. **Philippe N, Alcaraz JP, Coursange E, Geiselmann J, Schneider D.** 2004. Improvement of pCVD442, a suicide plasmid for gene allele exchange in bacteria. Plasmid **51:**246-255.
2. **Sun J, Li XP, Fang LX, Sun RY, He YZ, Lin J, Liao XP, Feng Y, Liu YH.** 2018. Co-occurrence of *mcr-1* in the chromosome and on an IncHI2 plasmid: persistence of colistin resistance in *Escherichia coli*. Int J Antimicrob Agents doi:10.1016/j.ijantimicag.2018.01.007.
3. **He Y-Z, Li X-P, Miao Y-Y, Lin J, Sun R-Y, Wang X-P, Guo Y-Y, Liao X-P, Liu Y-H, Feng Y, Sun J.** 2019. The IS*Apl1*$_2$ Dimer Circular Intermediate Participates in *mcr-1* Transposition. Frontiers in Microbiology **10**.
4. **Trebosc V, Gartenmann S, Royet K, Manfredi P, Tötzl M, Schellhorn B, Pieren M, Tigges M, Lociuro S, Sennhenn PC, Gitzinger M, Bumann D, Kemmer C.** 2016. A novel genome editing platform for drug resistant *Acinetobacter baumannii* revealed an AdeR-unrelated tigecycline resistance mechanism. Antimicrobial Agents and Chemotherapy doi:10.1128/aac.01275-16**:**AAC.01275-01216.
5. **Datsenko KA, Wanner BL.** 2000. One-step inactivation of chromosomal genes in *Escherichia coli* K-12 using PCR products. Proc Natl Acad Sci U S A **97:**6640-6645.
